# Enhancing Parenting Using AI: Exploratory Hackathon

**DOI:** 10.2196/68780

**Published:** 2025-12-17

**Authors:** Peter Woods, Stephanie Donohoe, Louise Turtle, Udit Agrawal, Joshua Humphriss, Niel Cordes, Nathan Hodson

**Affiliations:** 1 Warwick Medical School University of Warwick Coventry, England United Kingdom

**Keywords:** hackathon, mental health, parenting, psychiatry, large language models

## Abstract

**Background:**

Parenting skills programs are the primary intervention for conduct disorders in children. The Pause app enhances these programs by providing digital microinterventions that reinforce learning between sessions and after program completion. The potential of artificial intelligence (AI) in this context remains untapped. Hackathons have proven effective for health care innovation and can facilitate collaborative development in this space.

**Objective:**

We aimed to rapidly build AI-powered features in the Pause app to enhance parenting skills.

**Methods:**

We undertook a 1-day hackathon that included an ideation phase drawing on the Design Council’s double diamond framework and a development phase using microsprints based on agile and scrum approaches. The interdisciplinary participants included medical professionals, developers, and product managers.

**Results:**

Participants identified 3 core problems: generating age-appropriate distractions, receiving feedback on parenting efforts, and effectively using the journal function. During the solution phase, a wide range of options were explored, resulting in 3 key solutions: AI-assisted idea generation, a tool for summarizing parenting interactions, and a weekly journal roundup. During the development phase, participants completed 4 microsprints. Teams focused on 3 workstreams: building a “weekly roundup” module, creating an AI-based distraction generator, and developing a summarizer for active play sessions. These prototypes were integrated into the preproduction environment, with each workstream producing a functional component. Participant feedback (n=4) was unanimously positive, with all participants rating the event as “excellent” and highlighting the value of in-person collaboration.

**Conclusions:**

This 1-day hackathon used the double diamond approach to develop AI-powered features for parenting programs. Three solutions were explored across workstreams, resulting in 2 fully functioning and 1 near-functioning app component. The rapid problem-solving approach mirrors other health technology hackathons and highlights the untapped potential of AI in digital parenting support, surpassing traditional e-learning or video-based methods. This work suggests broader applications of AI-driven coaching in fields like social care. Despite a small team, the hackathon was focused and productive, generating relevant solutions based on prior engagement with parents and practitioners. Future research will assess the impact of the app’s AI-powered features on parenting outcomes.

## Introduction

### Background

Parenting skills programs are the first-line treatment recommended for conduct disorder, the most common mental health disorder of childhood [1,2]. The Pause app enhances parenting skills programs by providing digital support between parenting program sessions [3,4]. Specifically, the Pause app contains a series of digital microinterventions that map to the core skills parents learn; between sessions, parents are prompted to use these digital microinterventions to strengthen their skills and reflect on their success [3,5].

The rapid increase in available artificial intelligence (AI) tools provides opportunities and challenges for children and families. For instance, in the educational domain, concerns have been raised regarding the impact of large language models (LLMs) on academic integrity and critical thinking, highlighting the disruptive potential of AI [6] alongside its benefits. Simultaneously, although AI presents novel avenues for support, it has not yet been systematically leveraged to strengthen parenting skills programs, which are crucial for the child’s well-being.

Hackathons have previously provided an effective “stepping stone for health care innovation” by creating space for “blue sky thinking” and collaboration [7]. They are structured using design-thinking approaches to address specific pain points [8]. The Pause app provides a platform through which the potential of AI can be directed toward solving parenting challenges. This paper reports on an exploratory hackathon, the central theme of which was to rapidly co-design and prototype AI-powered features to enhance the support offered by the Pause app, thereby addressing specific parenting needs identified through prior user engagement.

### Objective

We conducted a hackathon with the aim of rapidly building AI-powered features to enhance parenting skills within the framework of the Pause app.

## Methods

### Theoretical Approach

This hackathon was designed in keeping with the best practices reported by Poncette et al [7]. The design approach used was the Design Council’s double diamond framework [9]. In this approach, participants come up with as many problems as possible and then narrow them down to the most important problems (the first diamond), then they come up with as many solutions as possible before narrowing down to the best solutions (the second diamond). The selected development approach was a series of microagile development sprints [10].

**Figure 1:**
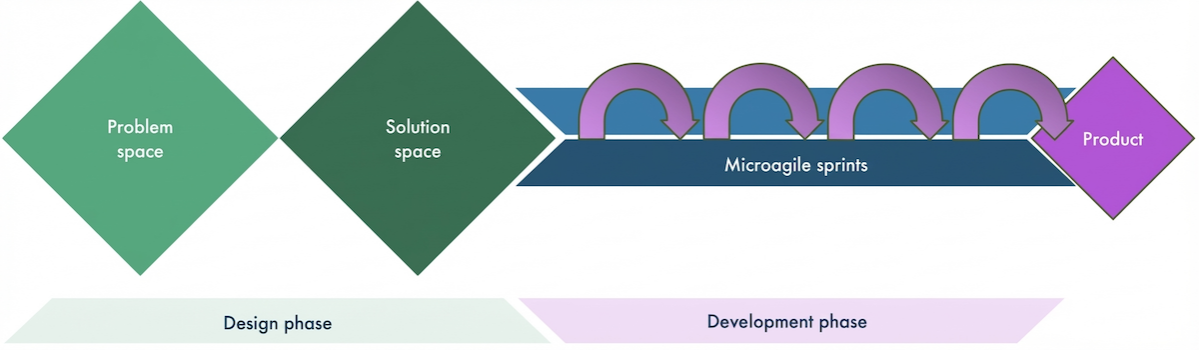
A diagrammatic illustration of the hackathon.

Figure 1 presents a schematic outlining the structure of the 1-day exploratory hackathon. The hackathon was designed to rapidly develop AI-powered features for the Pause parenting app and comprised a design phase using the Design Council’s double diamond framework, followed by an iterative development phase using microagile sprints.

### Community Building and Stakeholder Involvement

We undertook a bootstrapping-style hackathon, not a corporate-style event, to enable a small team to work on specific issues [7]. Participants in this hackathon were drawn from the digital mental health teams at the University of Warwick and several of them had previously worked together. Overall, 2 medical doctors, 3 developers, and 2 product managers participated.

### Hackathon Theme

The hackathon theme was set through development work with parents and parenting program practitioners who have used the Pause app [3,4]. The theme was announced to participants in advance. Three of the participants had been involved in analyzing the user data.

### Venue and Timing

The selected venue was a collaboration space at the University of Warwick, due to its available facilities for individual and group work. The development day took place on a Sunday so those with clinical and industry commitments in the usual working week could collaborate.

### Announcement

The hackathon was announced 1 month before the main event. Participants knew they would be building on the Pause app, and all participants had access to the Pause app before launch. All developers had access to the Pause development area before coding.

### Ethical Considerations

The hackathon activities described in this manuscript focused on formative development work by the study team and did not involve direct recruitment or new data collection from human subjects. However, the hackathon drew on anonymized patient and practitioner themes obtained from a previous foundational study [3], which had received ethics approval from the University of Warwick’s Biomedical Sciences Research Ethics Committee (BSREC 14/23-24). For that foundational study, informed consent was obtained from participants for their feedback to be used for improving digital mental health provisions, including the Pause app. For the hackathon itself, team members were invited to contribute based on their professional roles.

No new personal data were collected from hackathon participants. Data from the previous research were fully anonymized, and only nonidentifiable themes were used during the hackathon. Parents and practitioners from the foundational study were compensated with a US $32 voucher [3]. Hackathon team members contributed voluntarily as honorary fellows or as part of their compensated professional duties. No images in this manuscript identify individuals, and any future illustrative images will ensure anonymity or be accompanied by explicit consent.

### Hackathon Plan

The hackathon development day lasted from 10 AM to 6 PM with a design phase and a development phase. At 10 AM, there was a presentation that included icebreakers, inspirational talks, and the acknowledgment of participant expertise. Then, the 3 core problems areas were presented (idea generation, feedback on parenting encounters, and journal fatigue). Next, participants had a 30-minute group discussion to explore the problems in the widest sense before narrowing down on definitions of specific problems. Then, participants broke into smaller groups to consider the widest range of possible solutions, spending 1 hour on this. Another whole-group discussion was undertaken to select the most promising solutions.

The remainder of the day was broken into augmented microagile sprints [10]. Participants had 1 hour to conduct development microsprints during which time participants worked on different solutions. After every 60 minutes, a 5-minute microreview was conducted, where participants reviewed the progress of each other. Microreviews followed the agile scrum “stand-up” and “sprint review” approach of asking (1) what they had done; (2) what they were about to do; and (3) whether there were any barriers that needed to be unblocked [11].

### Lessons Learned and Follow-Up

The hackathon day ended with a presentation of the working code produced [10]. Functioning solutions were shared with all participants. Next steps for development were identified. Feedback was shared verbally and in writing, with space for a “miniretrospective” to improve future ways of working [11].

### Hackathon Process and Prototyping Outputs

#### Broadening the Problem Space

Three core ideas were presented: (1) parents need more ideas; (2) parents need more feedback; and (3) parents struggle with journaling. A wide range of problems were considered around these ideas.

#### Narrowing the Problem Space

Three problems were selected. The first was that parents find it hard to come up with age-appropriate distractions for their children. The second was that parents do not obtain feedback on their efforts to encourage their children. The third was that parents do not use the journal function effectively.

#### Broadening the Solution Space

A wide range of solutions were considered, some of which addressed multiple areas in the problem space. Participants considered adding new prompts for ways in which parents can spend time together with their children. They considered whether to provide real-time audio coaching to parents while they played with their child. Participants considered creating journal voice notes or journal entries through text and using these to create a coaching conversation with an AI chatbot. They also considered creating a function to summarize the content of time together and provide feedback. Participants considered allowing parents to select and rate from pregenerated ideas, generating prompts for parents, and providing parents with a weekly roundup of journaling activity over the week, inspired by Spotify Wrapped.

#### Narrowing the Solution Space

Participants settled on 3 solutions, one matching each of the initial problems. The first was AI-assisted idea generation for distracting children, the second was a tool to summarize and encourage time together with children, and the third was a weekly roundup to gamify the journaling process. These 3 ideas formed the basis for 3 workstreams during the development phase.

It maps the 3 core parenting challenges presented to participants, the specific problem definitions narrowed down during the first diamond of the Design Council's double diamond framework, examples of solutions explored, and the final AI-powered solutions selected for prototype development during the hackathon held at the University of Warwick.

### Development Phase

The development phase proceeded through four 1-hour microsprints covering the 3 workstreams. These are described in Table 1. The table details the specific tasks undertaken and outputs achieved by the interdisciplinary teams (medical doctors, developers, and product managers) in each workstream.

**Table 1:** Outcomes of development microsprints.

	Workstream 1	Workstream 2	Workstream 3
Microsprint 1	Team 1 mapped out a new “weekly roundup” module. It will pop-up every week, ideally the day before their next session. It will ask parents questions about what they think their main section of the app is. Then, it will also give them some data on their parenting. This was sketched, and no code was made.	Team 2 started making an Android APIa to pull information through to the app. Team 2 had some difficulty making it work, so they asked colleagues to identify the bug.	Team 3 made a “time together” summarizer, which can transcribe a short conversation and then summarize it, including positives and negatives.
Microsprint 2	Team 1 finished sketching out the module and prepared it in the development space. A new module was made within the app codebase.	Team 2 successfully made the API generate 5 personalized distractions based on a prompt word.	Team 3 made a Time Together summarizer which can listen to a conversation and then summarize it, including positives and negatives.
Microsprint 3	Team 1 started drafting code to pull together star ratings and gather parents’ thoughts.	Team 2 incorporated the AIb distraction generator into the distract module. Parents can add the AI-generated distractions into their list of distractions. Parents can delete distractions they do not like.	Team 3 incorporated the summarizer into the app so that when they hit play on “Time Together” it listens and then gives them feedback. Team strengthened the relevance of the parenting information.
Microsprint 4 (end of hackathon)	Team 1 mapped out the module flow and prepared the back-end code.	Team 2 added a “location” input as well as an “interests” input. This meant that the app generated highly targeted distractions for the situation people were in.	Team 3 completed a module where a time together session was summarized and positive and negative feedback were given. This feedback was then added directly to the journal and parents could add a star rating out of 5 for their experience.

^a^API: application programming interface.

^b^AI: artificial intelligence.

## Results

Of the 7 participants who attended the hackathon, 4 (57%) completed a postevent feedback survey. The results were unanimously positive and are presented in subsequent sections.

### Participant Ratings (Quantitative Results)

All respondents (4/4, 100%) rated the hackathon as “excellent.” Furthermore, all respondents agreed that it would be “worth doing another hackathon,” with comments, including “Yes!,” “Definitely,” and “When there’s a need for new features.”

### Participant Experience (Qualitative Results)

We analyzed qualitative feedback, which fell into 3 themes: positive aspects, suggestions for improvement, and ideas for future events.

#### Positive Aspects

The most valued aspects of the hackathon were in-person collaboration and rapid development. Comments included the following:

Being face to face.

Getting together to try out fresh new ideas.

Coming up with ideas and making them quickly.

#### Suggestions for Improvement

The main suggestion for future events was to have more time. One participant suggested it would be beneficial to do the following:

Have more than one day to try and get a fully working prototype and flesh out more ideas.

Test with users and do some iteration.

#### Ideas for Future Events

When asked about future events, participants suggested including more diverse expertise, such as,

Design...to think about interaction.

Combining it [the hackathon] with a conference.

Maybe lots of teams and prizes.

## Discussion

### Principal Findings

The primary objective of this study was to rapidly build AI-powered features to enhance parenting skills via the Pause app. This objective was successfully met through a 1-day hackathon, which resulted in the development of 2 fully functioning and 1 near-functioning app component designed to address core parenting challenges identified from a previous stakeholder engagement. These outcomes confirm that an intensive, structured hackathon approach—using frameworks such as the double diamond and agile microsprints—is a viable methodology for the swift creation and initial integration of AI-powered tools in this domain. This methodological success is further supported by our new quantitative and qualitative participant feedback (see the Results section), where all respondents (4/4, 100%) rated the event as “excellent” and highlighted the value of in-person, rapid collaboration for “coming up with ideas and making them quickly.” These prototypes are now prepared for subsequent testing and validation.

The AI-powered features developed during this hackathon offer potential advantages over traditional methods of delivering parenting skills support, such as static e-learning modules or periodic video calls. The AI-assisted idea generator for distractions offers a substantial step beyond static lists by providing suggestions tailored to the child’s age, with the underlying AI capability allowing for future enhancements incorporating more sophisticated contextual prompts, thereby reducing parental cognitive load in real time. The AI-powered summarization tool for parenting interactions, leveraging multimodal input (specifically audio analysis), provides parents with timely and specific feedback, and offers a more objective basis for self-assessment and reinforcement of positive parenting techniques—a feature not readily achievable with traditional non-AI tools. Furthermore, the AI-driven weekly journal roundup aims to combat journal fatigue and enhance reflection by transforming logged experiences into an engaging, summarized format, potentially identifying patterns and highlighting successes. These capabilities highlight the potential of AI to deliver more interactive, responsive, and individualized support, thereby deepening the engagement and impact of digital parenting interventions. Our exploration of rapidly prototyped AI features aligns with emerging evidence in this field. For instance, Entenberg et al [12] found that an AI-based chatbot microintervention for parents demonstrated meaningful engagement, learning, and efficacy, underscoring the promise of AI in delivering accessible parenting support. This paper builds on such insights by demonstrating a rapid and collaborative methodology—the hackathon—for developing a broader suite of AI-driven tools tailored to specific parenting challenges, such as idea generation for distractions and reflective journaling, thereby expanding the repertoire of potential AI applications within digital parenting platforms.

The development of these interactive and personalized features was facilitated by the selection of LLMs, favored for their advanced text generation, summarization, and multimodal audio analysis capabilities. Specifically, publicly available models accessed via their respective APIs (application programming interfaces), with versions current as of October 2024, were used. This included GPT-4o by OpenAI for text-based processing and Gemini 1.5 Pro by Google for its multimodal capabilities, particularly audio analysis for the summarization tool. This choice reflects the rapidly evolving landscape of accessible AI tools. The rapid problem-solving approach achieved in this 1-day event mirrors successes reported in other health technology hackathons [7,8], demonstrating an effective pathway from identified user needs to functional prototypes. The immediate measure of success for the prototypes developed within this hackathon was their functional integration into the preproduction environment of the Pause app. Formal preliminary user feedback sessions or expert evaluations were not conducted during the hackathon itself, but are planned as crucial next steps for development and validation.

Existing digital parenting interventions have primarily used e-learning or video calls [13]. This project suggests that current AI technology has much deeper implications for supporting parents, particularly within a platform like the Pause app. The availability of LLMs also has wider implications for mental health beyond parenting, such as improving therapy processes [14]. Indeed, the use of AI to support, coach, and encourage carers could have profound implications in various domains, for example, in adult social care, an area yet to be fully explored.

The integration of AI into parenting supports, particularly tools that analyze personal interactions or provide advice, raises important ethical considerations beyond the conduct of this specific hackathon. These include ensuring data privacy and security for sensitive family information processed by LLMs, obtaining informed consent from parents for AI analysis of their inputs, and mitigating potential biases in AI-generated advice. The psychological impact of AI-generated feedback, such as the risk of overreliance or misinterpretation leading to parental anxiety, also warrants careful consideration and the development of clear guidelines for responsible deployment, emphasizing AI as a supportive tool rather than a replacement for human judgment or professional guidance.

### Limitations

There are several limitations. The 1-day hackathon format, while fostering rapid development, inherently limits the depth of exploration and the complexity of the solutions that can be built. The development work with parents and parenting practitioners that preceded this hackathon meant we could prime participants to pursue relevant ideas; however, the small number of hackathon participant feedback (n=4), although conducive to a focused “bootstrapping” approach, may restrict the diversity of ideas and solutions generated compared to a larger event and introduces potential bias from a specialized group primarily from one institution. A larger, more diverse group of participants might generate a broader array of solutions but could also introduce logistical complexities in a single-day format. Furthermore, while this hackathon demonstrated technical feasibility, the integration of AI into sensitive contexts like parenting presents considerable challenges. These include ensuring the ethical use of data, managing user expectations, preventing overreliance on AI-generated advice, addressing potential biases in AI models, and ensuring equitable access for all parents, which were only touched upon thematically during this event and require ongoing dedicated research.

### Conclusions

The primary objective of this study, to rapidly build AI-powered features to enhance parenting skills via the Pause app, was successfully achieved. This 1-day hackathon resulted in the development of 2 fully functional and 1 near-functional app component, demonstrating the feasibility of this approach for swift prototyping and initial integration.

The key conclusions from this work are 2-fold: first, that intensive, structured co-design events similar to hackathons, using frameworks such as the double diamond and agile microsprints, serve as a valuable and efficient methodology for exploring and actualizing the potential of AI in applied digital health settings. Second, this exploration highlights that AI, particularly LLMs, offers novel avenues for enhancing digital parenting interventions, moving beyond traditional e-learning or video-based methods to provide more interactive and potentially personalized support.

We therefore recommend hackathons to other mental health teams considering the integration of AI into their digital platforms, as this format allowed our team to focus on underlying user problems and generate innovative solutions outside of routine work pressures. Future research will be essential to evaluate the uptake, user engagement, and ultimately the clinical impact of these AI-powered parenting support features on parenting skills and child outcomes.

## Abbreviations

AI: artificial intelligence
API: application programming interface
LLM: large language model

## Data Availability

The hackathon reported in this manuscript focused on the development of software prototypes and did not generate traditional research datasets. Inquiries regarding the conceptual outputs or specific aspects of the prototypes described can be directed to the corresponding author, and access may be considered on a case-by-case basis, subject to institutional policies and the stage of development.
